# Aberrant expression of the S1P regulating enzymes, SPHK1 and SGPL1, contributes to a migratory phenotype in OSCC mediated through S1PR2

**DOI:** 10.1038/srep25650

**Published:** 2016-05-10

**Authors:** Sathya Narayanan Patmanathan, Steven P. Johnson, Sook Ling Lai, Suthashini Panja Bernam, Victor Lopes, Wenbin Wei, Maha Hafez Ibrahim, Federico Torta, Pradeep Narayanaswamy, Markus R. Wenk, Deron R. Herr, Paul G. Murray, Lee Fah Yap, Ian C. Paterson

**Affiliations:** 1 Department of Oral Biology and Biomedical Sciences and Oral Cancer Research & Coordinating Centre, Faculty of Dentistry, University of Malaya, 50603, Kuala Lumpur, Malaysia; 2 Dept of Molecular Genetics, The Royal Devon and Exeter Hospital, Barrack Road, Exeter, EX2 5DW, United Kingdom; 3 Department of Oral surgery, Edinburgh Postgraduate Dental Institute, University of Edinburgh, Edinburgh, EH3 9HA, United Kingdom; 4 School of Cancer Sciences, University of Birmingham, Birmingham, B15 2TT, United Kingdom; 5 Department of Biochemistry, Yong Loo Lin School of Medicine, National University of Singapore, 117456 Singapore; 6 Department of Pharmacology, Yong Loo Lin School of Medicine, National University of Singapore, 117456 Singapore

## Abstract

Oral squamous cell carcinoma (OSCC) is a lethal disease with a 5-year mortality rate of around 50%. Molecular targeted therapies are not in routine use and novel therapeutic targets are required. Our previous microarray data indicated sphingosine 1-phosphate (S1P) metabolism and signalling was deregulated in OSCC. In this study, we have investigated the contribution of S1P signalling to the pathogenesis of OSCC. We show that the expression of the two major enzymes that regulate S1P levels were altered in OSCC: SPHK1 was significantly upregulated in OSCC tissues compared to normal oral mucosa and low levels of SGPL1 mRNA correlated with a worse overall survival. In *in vitro* studies, S1P enhanced the migration/invasion of OSCC cells and attenuated cisplatin-induced death. We also demonstrate that S1P receptor expression is deregulated in primary OSCCs and that S1PR2 is over-expressed in a subset of tumours, which in part mediates S1P-induced migration of OSCC cells. Lastly, we demonstrate that FTY720 induced significantly more apoptosis in OSCC cells compared to non-malignant cells and that FTY720 acted synergistically with cisplatin to induce cell death. Taken together, our data show that S1P signalling promotes tumour aggressiveness in OSCC and identify S1P signalling as a potential therapeutic target.

Oral squamous cell carcinoma (OSCC) remains a major world health issue and is particularly prevalent in India and South East Asia. More than 250,000 new cases are diagnosed each year and, despite advances in cancer therapy, approximately 50% of patients die within 5 years^1^. Patients are often given multimodal treatment comprising surgery, chemotherapy and radiotherapy^2^ but loco-regional recurrences, distant metastases and second primary tumours occur frequently and are responsible for the poor patient prognosis^1^. Whilst our understanding of the molecular basis for the development of OSCC is improving^3^, molecular targeted therapies are not in routine use and new approaches to manage the disease are urgently required.

Sphingosine-1-phosphate (S1P) is a bioactive lipid that is derived from its membrane-bound precursor, ceramide^4^. Ceramide is converted to sphingosine by the action of ceramidases and, subsequently, S1P is generated when sphingosine is phosphorylated by activated sphingosine kinases (SPHK1 and SPHK2). S1P can be dephosphorylated back to sphingosine by sphingosine phosphatase or irreversibly degraded by S1P lyase (SGPL1)^5^. The balance between S1P and its metabolic precursors, ceramide and sphingosine, the so called sphingosine rheostat, regulates cell fate with a shift towards ceramide inducing cell growth arrest and apoptosis, whereas S1P production promotes cell survival^6^. The effects of S1P are largely due to the binding to one or more of a family of five G-protein coupled receptors, termed S1PR1-5^7^, which then stimulate multiple signalling cascades^8^.

S1P is involved in a wide variety of cellular processes, such as proliferation, apoptosis, migration and angiogenesis^9^,^10^ and S1P can contribute to tumorigenesis^11^,^12^. In part, the cancer-promoting effects of S1P result from alterations in S1PR expression^9^,^11^,^13^,^14^. Although aberrant S1P signalling has been demonstrated in a number of human tumours^11^,^12^, there is limited information on the role of S1P in the pathogenesis of OSCC. SPHK1 expression has been reported to be upregulated in head and neck squamous cell carcinoma (HNSCC)^4^,^15^,^16^ and SPHK1-deficient mice were resistant to 4-nitroquinoline-1-oxide (4-NQO)-induced carcinogenesis^4^.

A number of specific agonists and antagonists of S1P signalling have been developed as research tools and potential therapeutics^9^. Notably, 2-amino-2-[2-(4-octylphenyl)]-1,3-propanediolhydrochloride (FTY720; fingolimod), an immunomodulatory drug has recently been approved for the treatment of relapsing multiple sclerosis. After phosphorylation, FTY720 binds to four of the S1PRs (S1PR1/3/4/5) and although FTY720 has an initial agonist activity on these receptors, it subsequently causes receptor internalization^17^,^18^,^19^,^20^. In addition to modulating the S1PRs, FTY720 can inhibit SPHK1 activity^21^ and activate the tumour suppressor, protein serine/threonine phosphatase type 2A (PP2A)^22^. Due to the pleiotropic properties of the drug, FTY720 can inhibit proliferation and migration of a variety of cancer cell lines, and promote their apoptosis and chemo-sensitivity. FTY720 also inhibits tumour growth, angiogenesis and metastasis *in vivo*^23^.

In this study, we examined the expression of SPHK1, SGPL1 and the S1PRs in OSCC tissues and determined the effects of S1P on OSCC-derived cell lines. We show that SPHK1 is significantly over-expressed in OSCC, whilst low levels of SGPL1 correlates with poor patient survival. We also show that S1P can protect OSCC cells from cisplatin-induced death as well as enhance their migration and invasion. Our data also show that S1P receptor expression is deregulated in primary OSCCs and that S1PR2 is over-expressed in a subset of tumours, which in part mediates S1P-induced migration of OSCC cells. Lastly we show that FTY720 induced significantly more apoptosis in OSCC cell lines compared to non-malignant cells. Taken together, our data show that S1P signalling promotes tumour aggressiveness in OSCC and that S1P inhibitors and/or sphingosine analogues have promise as novel therapeutic agents.

## Results

### Deregulation of the sphingosine rheostat in OSCC

**Figure 1:**
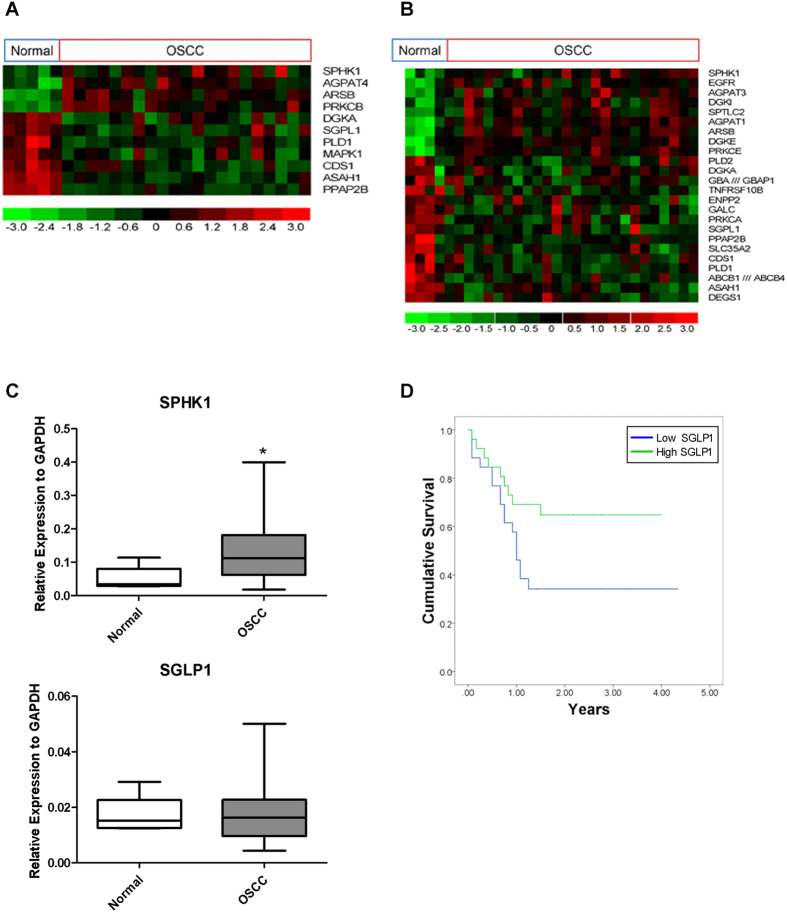
Dysregulation of genes involved in S1P signalling in OSCC patients. Heat-map of S1P-related genes differentially expressed (p < 0.05 and absolute fold change >1.4) between oral squamous carcinoma and normal oral mucosa in UK (**A**) and Sri Lankan (**B**) cohorts. Expression level of each gene was scaled to have mean value of 0 and standard deviation of 1 and represented by a green to red colour scale as shown at the bottom of the figure. (**C**) Box plots show expression of SPHK1 and SGLP1 in normal individuals and OSCC patients as determined by QPCR. Expressions are normalised to GAPDH. *ρ < 0.05 (Mann Whitney U test). (**C**) Kaplan Meier survival analysis was performed for OSCC samples with low and high expression of SGLP1. Lower expression of SGLP1 correlated with poor survival (ρ < 0.05).

Analysis of our own microarray data^24^ showed that the expression of a number of genes involved in sphingolipid metabolism and signalling were deregulated in OSCCs. There are 63 genes on the HG-Focus array that are associated with sphingolipid metabolism and S1P signalling (Table S1). Of these 63 genes, 11 and 24 genes were differentially expressed between OSCCs and normal oral mucosa from UK and Sri Lankan patients, respectively (Fig. 1A,B). Notably, the expression of SPHK1 was strongly up-regulated, whilst levels of SGPL1 were reduced in OSCCs compared to normal oral mucosa. Therefore, we examined the expression of SPHK1 and SGPL1 in an independent set of tumours (n = 52) and non-malignant control tissues (n = 5) by QPCR. The expression of SPHK1 was significantly up-regulated in the OSCCs compared to normal oral tissues (*ρ* < 0.05) (Fig. 1C). Although the mRNA levels of SGPL1 were not significantly different (ρ > 0.05) between the normal and malignant tissues (Fig. 1C), Kaplan Meier survival analysis revealed that low levels of SGPL1 mRNA correlated with a worse overall survival (Fig. 1D; ρ < 0.05). No significant difference in the overall survival was observed between OSCCs with low and high expression of SPHK1 (ρ > 0.05; data not shown). Taken together, our data from three independent cohorts of patients shows that the sphingosine rheostat is deregulated in OSCC and this deregulation has clinical significance.

### S1P protects OSCC cells from cisplatin induced death and promotes tumour cell migration and invasion

**Figure 2:**
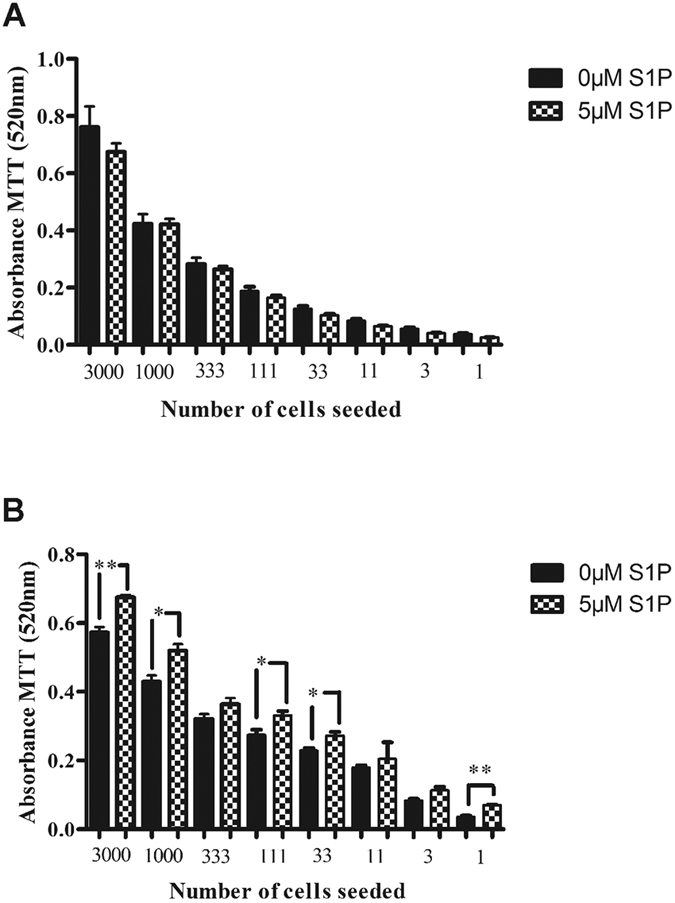
Effect of S1P on Cisplatin-induced apoptosis. H357 cells were either untreated (**A**) or were incubated for 3 hours with 1 μM Cisplatin (**B**) in serum free media in the presence or absence of 5 μM S1P. Twenty four hours later the cells were harvested, and replated in a 96-well plate in complete media using 1 in 3 serial dilutions. After 7 days in culture, an MTT assay was performed. Graphs show the mean ± standard deviation of three experiments performed in triplicate. **P < 0.05, ***P< 0.001 (Unpaired t-test).

We next investigated the phenotypic impact of S1P on OSCC cell lines *in vitro*. We used clonogenic assays to evaluate the effect of S1P on long-term survival in response to the chemotherapeutic drug, cisplatin, commonly used to treat OSCC patients. Whilst treatment with S1P alone had no effect on the clonogenic growth of H357 cells (Fig. 2A), S1P caused a significant increase in the clonogenic survival of cells treated with cisplatin (Fig. 2B).

**Figure 3:**
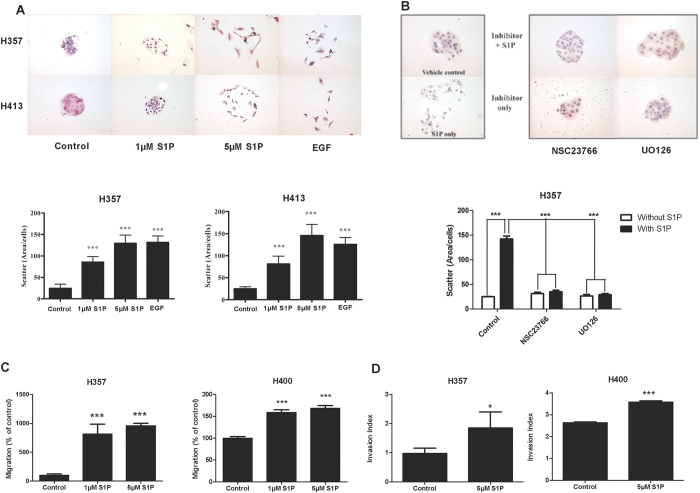
Effect of S1P on cell migration and invasion. Mitomycin C-treated colonies in serum free media were either left untreated or treated with S1P (1 and 5 Μm); (**A**) or with 5 μM S1P in the presence or absence of chemical inhibitors of Rac-1 (NSC23766; 5 μM) or MEK1/2 (UO126; 10 μM) (**B**) for 48 hours. Epithelial growth factor (EGF) was used as a positive control of cell scatter. Representative images from three independent experiments are shown. The extent of cell scattering was analysed by measuring the density of colonies of H357 and H413 cells (cells/μm^2^). The data represents the average of measurements taken from thirty colonies chosen at random. (**C**) Migration was measured using transwell assays using H357 and H400 cells in the absence or presence of 1 or 5 μM S1P. Results are expressed as percentage of migrated cells in the untreated control (=100%) ± SD. (**D**) Invasion assay was performed by plating H357 or H400 cells on collagen in the presence or absence of 5 μM S1P. Results represent data obtained from three independent experiments. ***ρ < 0.001, **ρ < 0.01 and *ρ < 0.05 (Dunnett’s post-hoc test or unpaired T-test).

We next examined the influence of S1P on the motility, migration and invasive capacity of OSCC cells. In colony dispersal assays, treatment of established colonies with 1 μM S1P induced loss of cell-to-cell adhesion as cells visibly dissociated and became less tightly clustered. Treatment with 5 μM S1P caused the colonies to disperse into single cells that had migrated away from the original centre of the colony (Fig. 3A). Quantification of colony scatter confirmed a highly significant increase in cell motility following treatment with either 1 μM or 5 μM S1P (Fig. 3A; *ρ* < 0.001). S1P-induced cell motility was greatly attenuated in the presence of inhibitors of, Rac1 and MEK 1/2 (Fig. 3B; *ρ* < 0.001), suggesting activation of multiple pathways downstream of the S1P receptors are involved in S1P-induced migration and cell motility. Similarly, S1P significantly enhanced the migration of OSCC cells in transwell assays (Fig. 3C) and promoted tumour cell invasion in collagen invasion assays (Fig. 3D).

### S1PR2 is over-expressed in OSCCs and mediates S1P-induced cell migration

**Figure 4:**
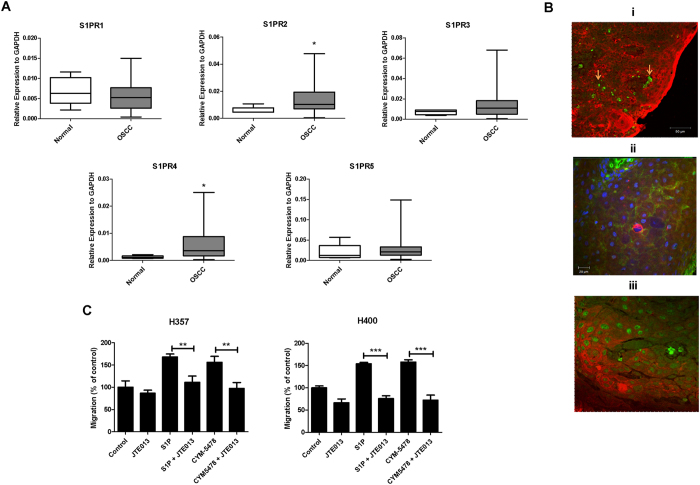
S1PR expression and role of S1PR2 in migration. (**A**) Box plots show expression of each S1PRs in normal individuals and OSCC patients as determined by QPCR. Expressions are normalised to GAPDH. *ρ < 0.05 (Mann Whitney U test). (**B**) Immunohistochemical evaluation of S1PR2 expression in normal and malignant tissues. Tissues were dual stained with pan-cytokeratin (Cy3, red) and S1PR2 (flourescein, green) antibodies. i. Normal tonsil showed weak nuclear staining in the stratified epithelium; occasionally moderate nuclear staining was also observed in normal epithelium. Red blood cells within the epithelium stained strongly for S1PR2 (arrows) and served as positive internal controls. ii and iii. Two separate well-differentiated OSCCs showing strong S1PR2 staining. The staining was cytoplasmic and focally membranous (ii) or nuclear (iii). (**C**) Transwell assays were performed in the presence or absence of 5 μM S1P, 5 μM JTE013 and/or 5 μM CYM5478 in the lower chamber. Results are expressed as percentage of migrated cells in the untreated control (=100%) ± SD. ***ρ < 0.001 and **ρ < 0.01 (unpaired T-test).

We next explored which S1P receptors might mediate the effects of S1P on OSCC cell migration. QPCR analyses showed that the expression of the S1PRs was heterogeneous and the majority of primary OSCCs expressed all five receptors. S1PR2 and S1PR4 were significantly upregulated in OSCCs compared to normal oral mucosa (ρ < 0.05) (Fig. 4A). The Kaplan Meier survival distributions for low and high expression of the individual S1PRs were not statistically significantly different (ρ > 0.05; data not shown). As S1PR2 has been more strongly implicated in carcinogenesis, the expression and cellular localisation of S1PR2 protein were examined in 41 formalin fixed, paraffin embedded primary OSCCs and 3 non-neoplastic tonsil samples used as a source of normal squamous epithelium. We did not include morphologically ‘normal’ epithelium adjacent to the tumours in our analysis because of potential ‘field’ effects. 32 out of 41 (78%) OSCCs demonstrated S1PR2 staining which was membranous, cytoplasmic and/or nuclear (Fig. 4B). We showed that while there was weak/moderate staining in normal tonsillar epithelium (mean H-score of 68; range 5–120; Fig. 4B), S1PR2 was over-expressed (H-score >120; range 120–285) in 16 of 41 tumours (39%). To investigate the functional role of S1PR2, we took advantage of the S1PR2 agonist, CYM5478, and the S1PR2 antagonist, JTE013, to examine cell migration *in vitro*. These compounds were shown to be specific using a TGF-α shedding assay that measures GPCR activation (Herr *et al.*, manuscript under review)^25^. The cell lines used (H357 and H400) expressed all S1P receptors (Figure S1). JTE013 reduced S1P-induced migration of H357 and H400 cells, while CYM-5478 induced migration of these cells, an effect which could be blocked by JTE013 (Fig. 4C), thereby demonstrating that the pro-migratory effects of S1P on OSCC cells are mediated, at least in part, by S1PR2.

### FTY720 inhibits the viability and clonogenic survival of OSCC cells by inducing apoptosis

**Figure 5:**
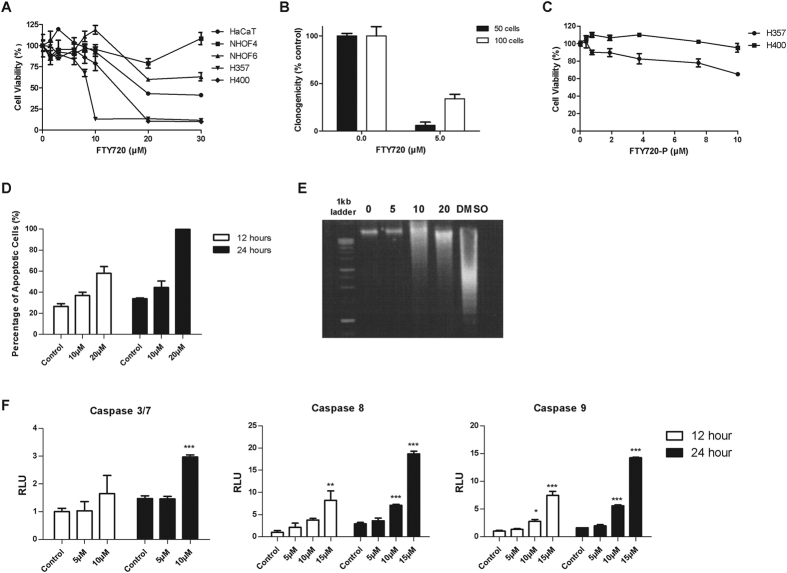
*In vitro* cytotoxic effects of FTY720. (**A**) The dose-dependent effect of FTY720 in normal oral fibroblasts (NHOF4 and NHOF6), normal keratinocytes (HaCaT) and OSCC (H357 and H400) cell lines. Cells were treated with FTY720 in complete media for 72 hours. Results are expressed as a percentage of viability of cells treated with vehicle control ( = 100%) and are shown as mean ± SD values of triplicates. (**B**) H400 cells (50 or 100 cells) were seeded and cultured in complete media for 24-hours and then treated with FTY720 (5 μM). The results represent the mean number of colonies (>50 cells) formed ± SD in three separate dishes for each condition and are expressed as a percentage of colonies formed by cells treated with vehicle control (=100%). (**C**) The cytotoxic effect of FTY720-P in H357 and H400 cells using MTT assays. Results are expressed as a percentage of viability of cells treated with vehicle control (=100%) and are shown as mean ± SD values of triplicates. (**D**) H400 cells were treated with vehicle control, 10 or 20 μM FTY720 in complete media. Results show the percentage of total apoptotic cells (early and late) ± SD of duplicates as determined by Annexin V/PI dual staining. (**E**) DNA laddering observed for H400 cells treated with 10 μM and 20 μM of FTY720 for 48 hours. Cells treated with 5% DMSO were used as a positive control. (**F**) H400 cells were treated at different concentrations of FTY720 in complete media for 24 hours, prior to measuring caspase 3/7, 8 and 9 activities. The results are expressed as relative luminescence units (RLU) calculated based on the luminescence produced relative to cells treated with vehicle control at 12 hours (=1). *ρ < 0.05 and **ρ < 0.01 (Dunnett’s post-hoc test).

As we showed that the sphingosine rheostat and S1PR expression are deregulated in OSCC, we examined the cytotoxicity of FTY720 on two OSCC cell lines (H357 and H400), one immortalised normal keratinocyte (HaCaT) and two normal oral fibroblasts (NHOF4 and NHOF6) using MTT assays. FTY720 demonstrated selective cytotoxicity with a lower IC_50_ in the OSCC cells (approximately 10 μM) compared to HaCaT (>20 μM) and the NHOFS (>30 μM; (Fig. 5A)). In addition, FTY720 significantly impaired the clonogenicity of H400 cells (ρ < 0.001; Fig. 5B). As FTY720 can be phosphorylated by SPHK2 to generate FTY720-P, a modulator of all S1PRs except S1PR2^26^, we sought to determine whether the effect of FTY720 on the cell survival of OSCC cells is S1PR-dependent or -independent. H357 and H400 cells expressed SPHK2 (Figure S1) indicating that these cells can phosphorylate FTY720. Treatment of H400 with FTY720-P had no effect on cell viability (400 nM-10 μM), whilst in H357, FTY720-P had no effect at 400 nM but caused a dose dependent reduction in cell viability at higher concentrations (35% reduction at 10 μM) (Fig. 5C).

Flow cytometric analysis and apoptotic DNA laddering demonstrated that FTY720 induced cell death through apoptosis. Treatment of H400 with 10 and 20 μM of FTY720 for 12 and 24 hours increased both early and late apoptotic cell populations in flow cytometry analysis (Fig. 5D). FTY720 also induced DNA fragmentation in H400 cells (Fig. 5E). The apoptosis induced by FTY720 was concomitant with the activation of caspase-3/7, -8 and -9, indicating that this drug activates both extrinsic and intrinsic apoptotic pathways (Fig. 5F).

### FTY720 inhibits ERK and Akt phosphorylation

**Figure 6:**
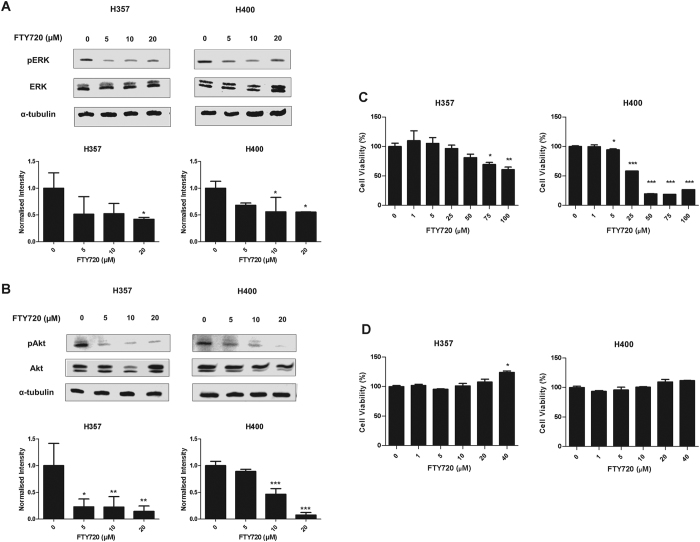
FTY720 induces ERK and Akt deactivation. Images show the inhibition of ERK (**A**) and Akt (**B**) phosphorylation in H357 and H400 cells following treatment with increasing concentrations of FTY720 (μM) in complete media for 24 hours. The intensity of the bands are normalized to α-tubulin and expressed relative to the cells treated with vehicle control (=1). Results are shown as mean ± SD values of three independent experiments. H357 and H400 cells were treated with either PI3K inhibitor (LY294002) (**C**) or MEK1/2 inhibitor (UO126) (**D**) in complete media prior to MTT assays. Results are shown as mean ± SD values of triplicates. *ρ < 0.05, **ρ < 0.01 and ***ρ < 0.001 (Dunnett’s post-hoc test).

To investigate the downstream pathways through which FTY720 induces apoptosis, we examined its effects on ERK and Akt signalling. FTY720 inhibited both ERK and Akt pathway activation in both H357 and H400 cells (Fig. 6A,B). To further elucidate the significance of these pathways in FTY720 induced apoptosis, we treated both H357 and H400 cells with increasing concentrations of phosphatidylinositol 3-kinase (PI3K) (LY294002) and MEK1/2 (UO126) inhibitors for 24 hours prior to MTT assay. The inhibition of ERK and Akt phosphorylation by UO126 and LY294002 was confirmed by Western blotting (Figure S2). LY294002, but not UO126, induced cell death in a dose-dependent manner (Fig. 6C,D), suggesting that the inhibition of Akt by FTY720 might be responsible for its effects on cell survival. However, expression of constitutively active Akt did not rescue the cells from the cytotoxic effects of FTY720 (Figure S3), suggesting that other pathways are also involved in FTY720-induced apoptosis in OSCC cells.

### Combination of FTY720 and cisplatin shows enhanced cell killing in OSCC cells

**Figure 7:**
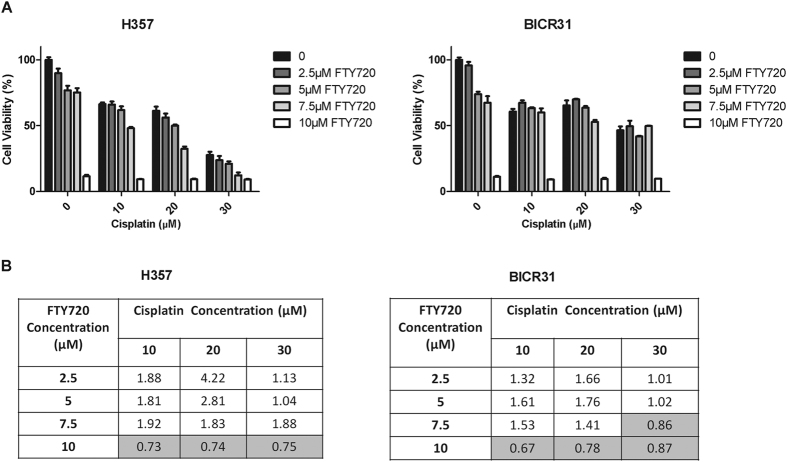
Combined effects of FTY720 and Cisplatin. (**A**) H357 and BICR31 cells were treated with various concentrations of FTY720 and Cisplatin in complete media for 72 hours prior to MTT assay. Graphs represent three independent experiments performed in triplicates. Percentage of cell viability is expressed relative to cells treated with medium containing diluent (DMSO) alone. (**B**) Combination index (CI) between FTY720 and Cisplatin was computed and the CI values obtained for each drug combination are shown in the table. Combinations with CI values lower than 1 (synergism) are highlighted in grey.

We next tested the effect of FTY720 on the sensitivity of OSCC cell lines to cisplatin. Cells were treated with cisplatin alone (0–30 μM), FTY720 alone (0–10 μM) or a combination of these two drugs in a relatively cisplatin-resistant (BICR31) and sensitive (H357) OSCC cell line prior to MTT assays (Fig. 7A). Drug combination analysis showed that synergism between FTY720 and cisplatin only existed at high FTY720 concentration (10 μM) (CI < 1) (Fig. 7B).

## Discussion

OSCC is a lethal disease with a 5-year mortality rate of around 50%. Molecular targeted therapies are not in routine use and novel therapeutic targets are required. There is now a substantial body of evidence to show that the expression of genes involved in the synthesis and breakdown of S1P is deregulated in tumorigenesis^12^. We show that the expression of SPHK1 is upregulated in OSCC, results that are consistent with previous reports in a variety of cancer types^27^, including other head and neck carcinomas^4^,^15^,^16^. We did not find any correlation with SPHK1 mRNA levels and patient survival and/or prognosis of OSCC patients or cancer stage, although such associations have been reported previously^15^,^16^. In our cohort of 52 patients, lower expression of SGPL1 correlated with poor survival of OSCC patients. SGPL1 levels have previously been reported to be reduced in colon and prostate cancers compared to normal tissues and this is linked to poor patient prognosis^28^,^29^. Taken together, our results demonstrate for the first time that both the synthesis and catabolism of S1P is deregulated in OSCC, which would lead to increased production of S1P. Therefore, we measured S1P levels in plasma from a small group OSCC patients and normal individuals using LC-MSMS to determine if circulating S1P levels might be a useful biomarker. However, there were no significant differences between the groups (Figure S4), indicating that elevated levels of S1P are likely to be localised within the tumours and surrounding tissues.

Having shown that OSCCs are likely to produce elevated amounts of S1P resulting from the deregulation of SPHK1 and SGPL1 expression, we examined the effect of exogenous S1P on the behaviour of OSCC-derived cell lines *in vitro*. S1P enhanced the survival of OSCC cells in response to the chemotherapy drug, cisplatin, consistent with studies showing an association of SPHK1 levels with poor response to chemotherapy^30^,^31^,^32^. Additionally, we also showed that S1P induced migration and invasion of OSCC cells. Collectively, our data show that the S1P produced by tumour cells would lead to a more invasive and aggressive phenotype. The biological response to S1P is thought to be altered in cancer because of an altered profile of S1PR expression on malignant cells^11^. We examined the expression of the S1PRs in OSCC for the first time and we demonstrated that S1PR2 and S1PR4 were significantly upregulated in primary OSCCs. We opted to validate the expression of S1PR2 by immunohistochemistry because this receptor is thought to be more involved in cancer pathogenesis and we confirmed that the S1PR2 was protein was elevated in 39% of tumours. This has functional significance because we showed that activation of this receptor promotes the migration of OSCC cells. Interestingly, we detected S1PR2 expression in the membrane, cytoplasm and nucleus of tumour cells and it has been shown recently that S1PR4 functions to prevent nuclear accumulation of S1PR2 to enhance the growth of ER negative breast cancer cells^33^. As we showed that S1PR4 is also over-expressed at the mRNA level in OSCCs, the functional cooperativity of these receptors in the pathogenesis of OSCC clearly warrants investigation.

Our data showed there was heterogeneity in the expression of the different S1PRs and when we classified the expression of the individual S1PRs into high or low expression, 29 of 52 (55%) of the OSCCs showed relatively high expression of three or more of the receptors (Figure S5), suggesting that other receptors might mediate some of the pro-tumorigenic effects of S1P. Interrogation of RNASeq data from a large (>500 tumours) HNSCC Cancer Genome Atlas data set also revealed a general deregulation of S1PR expression with approximately 30% of tumours showing up-regulation (1.5 fold) of one or more of the S1PRs^34^,^35^. These data indicate that there may be some redundancy between the receptors in mediating the pro-tumorigenic effects of S1P. Indeed, the S1PR1/3 antagonist, VPC23019, significantly inhibited S1P-induced OSCC cell migration (Figure S6), consistent with other reports in different tumour types^36^,^37^,^38^,^39^,^40^.

A number of strategies have been proposed to inhibit the pro-oncogenic effects of S1P. We opted to use FTY720 because this drug exhibits pleiotropic effects on the SPHK1-S1P-S1PR axis and affects various aspect of cancer pathogenesis^23^. We show that FTY720 selectively induced cytotoxicity in OSCC cells compared to non-transformed epidermal human keratinocytes (HaCaT) and normal human oral fibroblasts. Similar results have been reported in other cancer types^41^,^42^,^43^,^44^,^45^,^46^. FTY720 induced both caspase-8 and -9 activation in OSCC cells, indicating that this drug is involved in both extrinsic and intrinsic apoptotic pathways, results that are similar to those in multiple myeloma and chronic myelogenous leukemia cells^47^,^48^. We also show that FTY720 synergizes with the chemotherapy drug, cisplatin, in inducing cell death in OSCC cell lines. FTY720 has been shown to increase the cisplatin sensitivity of lung cancer cell through the activation of PP2A^49^ and enhance cell death in combination with cisplatin by inhibiting the Akt and epidermal growth factor (EGF) pathways in melanoma cells^50^.

In the present study, FTY720-P had no effect when used at 400 nM, a dose which is known to modulate the S1PRs^26^, which suggests that the effects of FTY720 are independent of the S1PRs. The cytotoxicity of FTY720-P in H357 cells at higher concentrations is most likely due to the de-phosphorylation of the drug to FTY720^51^,^52^. FTY720 treatment of OSCC cells resulted in the inhibition of Akt phosphorylation, although the effects of the drug could not be rescued by the ectopic expression of constitutively active Akt. The mechanisms involved in mediating the effects of FTY720 in OSCC cells remain to be determined but could include the inhibition of SPHK1 and/or activation of PP2A, amongst others^23^. In our study, treatment of cells with the SPHK1-selective inhibitor PF-543 (0.05–10μM; 72 hours) did not reduce the viability of OSCC cells (data not shown), in agreement with a previous report using HNSCC cells^53^. However, PF-543 treatment did not result in an accumulation of ceramide in HNSCC cells^53^, so a more comprehensive analysis with different SPHK1 inhibitors is required to conclusively investigate the effects of SPHK1 inhibition in OSCC cells.

In conclusion, we show for the first time, using three independent cohorts of patients, that the synthesis and catabolism of S1P is deregulated in OSCC, which would lead to an accumulation of S1P in the tumour and the associated microenvironment. We demonstrate that S1P contributes to a more motile and invasive phenotype in OSCC cells and that there is a general deregulation of the S1PRs in OSCC, with a subset of tumours over-expressing S1PR2 at both the mRNA and protein level. Lastly, we found that FTY720 induces selective apoptosis in OSCC cells compared to normal and non-transformed cells and that the drug might synergise with cisplatin in inducing cell death. Collectively, our results highlight the potential of targeting S1P and its signalling pathways in OSCC patients.

## Methods

### Materials

Sphingosine-1-phosphate (S1P; D-erythro S1P; Avanti Polar Lipids, Alabaster, USA) was dissolved in 95% methanol, dried under a stream of nitrogen gas and stored at −20 °C in aliquots of 100 nmol. Prior to use, S1P was constituted in 4 mg/ml fatty acid-free human albumin (FAFA; Sigma). Cisplatin [cis-Diammineplatinum(II) dichloride] and JTE013 [1-[1,3-Dimethyl-4-(2-methylethyl)-1H-pyrazolo[3,4-b]pyridin-6-yl]-4-(3,5-dichloro-4-pyridinyl)-semicarbazide] were obtained from Tocris Biosciences (Bristol, UK). FTY720 (2-Amino-2-(2-(4-octylphenyl)ethyl)propane-1,3-diol, HCl) and VPC23019 (2-Amino-N-(3-octylphenyl)-3-(phosphonooxy)-propanamaide) were obtained from Merck Millipore (Darmstadt, Germany) and R&D Systems (Minneapolis, USA), respectively. CYM-5478 (catalog #EN300-57094) was obtained from Enamine LLC. UO126 and LY294002 are from Merck Millipore and Sigma, respectively.

### Analysis of microarray data

Microarray raw data of oral squamous cell carcinoma in data set GSE51010 were downloaded from GEO. Probe level quantile normalisation^54^, RMA (robust multi-array analysis)^55^,^56^ and MAS5 detection analysis were performed using the affy package of the Bioconductor project (http://www.bioconductor.org). Fold change and p value were calculated using limma^56^. Probe set annotation was from Affymetrix “HG-Focus.na35.annot.csv”. Probe sets with “Negative Strand Matching Probes” were removed. Differentially expressed genes were identified using the criteria of absolute fold change >1.4, p value <0.05 and number of samples with “Present” calls > = 3. Gene expression heatmap was generated using dChip^57^.

### Cell lines, tissues and plasma samples

Details and culture conditions for the normal human oral fibroblasts (NHOFs) and OSCC cell lines have been described previously^58^,^59^,^60^. The non-tumorigenic keratinocyte cell line HaCaT^61^ were cultured in Dulbecco’s modified Eagle’s medium (DMEM) containing 10% fetal bovine serum (FBS).

Biopsies of OSCCs and samples of normal oral mucosa, plus plasma samples from OSCC patients and control healthy individuals, were obtained from the Malaysian Oral Cancer Database and Tissue Bank System (MOCDTBS) managed by the Oral Cancer Research and Coordinating Centre (OCRCC), University of Malaya. Information regarding the age, gender, cancer stage and survival status are shown in Table 1. Ethical approval for this study was obtained from the Medical Ethics Committee, Faculty of Dentistry, University of Malaya (DF OB1403/0009L).

### Quantitative real-time polymerase chain reaction (QPCR)

Total RNA was extracted using an NucleoSpin® RNA II kit (Macherey-Nagel, Düren, Germany) and subjected to reverse transcription using a High-Capacity cDNA Reverse Transcription kit (Applied Biosystems, Life Technologies, California, US). QPCR was performed in triplicate using the 7500 Fast Real-Time PCR system (Applied Biosystems) and TaqMan^®^ Gene Expression Assays (SPHK1: Hs00184211_m1; SGPL1: Hs00187407_m1; S1PR1: Hs001922614_s1; S1PR2: A139R4J; S1PR3: Hs00245464_s1; S1PR4: Hs02330084_s1; S1PR5: Hs00928195_s1) (Applied Biosystems). GAPDH (4326317E) (Applied Biosystem) was amplified in the same reaction to serve as an internal control for normalization. Fold changes in gene expression were measured using the comparative threshold cycle method (ΔΔCt).

### Liquid chromatography (LC) and mass spectrometry (MS)

Long chain base phosphates (LCB-P) were extracted and derivatised, as described previously^62^. 10 μl of plasma were mixed with 90 μl of methanol containing the internal standard, D-erythro-Sphingosine-1-phosphate (13C2D2–S1P; Toronto Research Chemicals) at a concentration of 20 ng/ml. The solution was sonicated for 30 min at room temperature and centrifuged for 10 minutes at 14000 g. The supernatant was dried and resuspended in 100 μl methanol for derivatization. TMS (10 μl) was added and the reaction incubated on a thermomixer for 20 min at RT. After centrifuging for 10 mins at 14,000 g the supernatant was dried. D-erythro-Sphingosine-1-phosphate (13C2D2–S1P; Toronto Research Chemicals) was used as an internal standard.

All LC-MS/MS experiments were performed using Agilent 1290 UHPLC connected to Agilent 6495 mass spectrometer. The column used was a Waters BEH HILIC 1.7 μm, 2.1 × 100 mm, kept at 60 °C during the analysis. The mobile phases were: 95% acetonitrile in 25 mM ammonium formate pH 4.6 and 50% acetonitrile in 10 mM ammonium formate pH 4.6 for A and B respectively. The pH was adjusted with formic acid. The gradient used was 99.9% to 40% A from 0 to 5 min, to 10% A from 5 to 6.5 min and equilibrated at 99.9% from 6.6 to 9 min. The flow used was 0.4 ml/min. The Agilent 6495 triple quadrupole (QQQ) mass spectrometer was operated in positive mode for MRM, electrospray voltage was set to 3500 V (Vcap), gas temperature 200 °C, gas flow 12 l/min, sheath gas temperature 400 °C. In positive ion MRM mode and with collision energy of 29 V, two product ions were monitored after CID of the S1P precursors. M/z 60 was used as a ‘quantifier’ and m/z 113 was used as a ‘qualifier’, as reported previously^62^. These ions were present after fragmentation of all the derivatised LCB-P species. Quantification was performed according to the internal standard method, comparing peak areas of the sample to the internal standard. All the samples were resuspended in 100 μl of mobile phase A before injecting 2 μl of sample for LC-MSMS analysis.

### Immunohistochemistry

Expression of S1PR2 and cytokeratin in primary OSCC tissue samples and non-malignant tonsils was determined by dual immunohistochemistry using Opal^TM^ TSA Plus Cyanine3/ Fluorescein immunofluorescence staining (NEL753001KT, Perkin Elmer). Primary cytokeratin AE1/AE3 antibody (mouse mAb, 1:1000 dilution, Dako) was used with anti-mouse IgG (goat) HRP-conjugated secondary antibody prior to signal amplification with TSA Plus Cyanine 3. The primary-secondary-HRP complex was then removed by microwave treatment. The staining procedure was repeated using primary SIPR2 antibody (rabbit pAb, 1:400 dilution, Sigma), anti-rabbit IgG (goat) HRP-conjugated secondary antibody and TSA Plus Fluorescein for detection. Immunohistochemical staining was semi-quantitatively evaluated using the H-score method. The percentage of tumours corresponding to an ordinal intensity value (0 = none, 1 = weak, 2 = moderate, 3 = strong) was assigned using whole sections. The H-score was defined as the sum of the percentage of tumour cells staining multiplied by the intensity level, resulting in a score ranging from 0 (no staining in any of the cells) to 300 (strong staining in all cells).

### Colony dispersal assay

1 × 10^3^ cells were seeded into 60-mm dishes for 96 hours until compact colonies of cells were formed and then incubated in serum free media for a further 48 hours. The cells were subsequently treated with mitomycin C (10 ug/ml) for 2 hours followed by the addition of S1P (1 μM or 5 μM) in the presence or absence of the Rac-1 inhibitor (NSC23766; 5 μM) or the MEK 1/2 inhibitor (UO126; 5 μM). After 72 hour incubation, the cells were fixed with formal saline and stained using haematoxylin and eosin (H&E). Stained cells were viewed using an Olympus CX-41 light microscope. Ten images were taken from each dish and the average area of scatter was calculated using Analysis software (Soft Imaging Systems) by drawing the smallest circle possible around the colonies of cells. The area of the circle was divided by the number of the cells within the circle to give the area relative to the number of cells. 5 μM epidermal growth factor (EGF) was used as a positive control of cell scatter.

### Transwell migration assay

Polycarbonate filters (8 μm pore size; Transwell, Corning, USA) were coated with 10 μg/ml fibronectin and placed into 24-well plates (Costar, Corning) to create upper and lower chambers. Cells were treated with 10 μg/ml Mitomycin C for 2 hours following serum-stravation for 24 hours. Cells were then resuspended in migration buffer (DMEM/F12 containing 0.25 mg/ml FAFA). 5 × 10^5^ cells were seeded into the upper chamber and allowed to migrate for 20 hours in the presence of the desired treatment or the corresponding vehicle control (prepared in migration buffer) in the lower chamber. Migrated cells were stained with 0.1% crystal violet (in 20% methanol) and counted in five random fields under 20x magnification.

### Invasion assay

Invasive ability of OSCC cells in response to S1P were examined by modifying protocols described previously^63^. Cells were serum-starved for 24 hours and treated with 10 μg/ml Mitomycin C for 2 hours prior to the experiment. Collagen gels were prepared on ice by mixing 4 ml of 3.75 mg/ml type 1 collagen from rat tail tendon (BD Biosciences, Oxford, UK), 5 ml of DMEM/F12 medium containing S1P (5 μM) and 1 ml 0.125 M NAOH/0.26M NaHCO_3_. The collagen mixture was then aliquoted into 24-well plates (500 μl /well) and allowed to solidify for 3 hours at 37 °C. 5 × 10^5^ OSCC cells were plated on top of the collagen surface and allowed to invade for 72 hours. The gel surfaces were then treated with trypsin for approximately 20 minutes and the number of trypsinised cells was counted. The collagen gels were melted by heating at 50 °C and the number of cells in the collagen was counted. The invasion index was obtained by dividing the number of cells in the gel (invaded cells) by the number of the cells in trypsin (non-invaded cells).

### Cell viability assay

1 × 10^4^ cells/well were seeded in 96-well plates and incubated at 37 °C for 24 hours. The cells were then treated with drugs, prior to the addition of 3-(4,5-dimethylthiazol-2-yl)-2,5-diphenyl tetrasodium bromide (MTT; Calbiochem, Merck Millipore; 2 mg/ml). Following 4 hours of incubation with MTT, 10% sodium dodecyl sulphate (SDS) in 1 mM hydrochloric acid were added into the wells to dissolve the blue crystal. After overnight incubation, cell viability was assessed by measuring the absorbance at 575 nm with a reference wavelength of 650 nm. The IC50 and the combination index (CI) (for drug combination analysis) were computed by using CompuSyn software (ComboSyn, Inc., Paramus, US). Based on the mass-action law, this software predicts synergism (CI < 1), antagonism (C1 > 1) or mere additive effect (C1 = 1) when the drugs are combined for treatment.

### Clonogenic assays

Two different protocols were used for clonogenic assays. For Cisplatin treatment, 1 × 10^6^ cells were seeded into 100-mm dishes for 24 hours and then treated with 5 μM S1P for 90 minutes, before the addition of Cisplatin (1 μM), or vehicle control (DMSO). After 3 hour incubation, the cells were harvested by trypsinisation and replated into 96-well plates using 1 in 3 serial dilutions from 3000 cells per well to 1 cell per well. After 7 days in culture, viable cells were assessed by MTT assays. For FTY720 treatment, 50 or 100 cells were seeded in 60-mm dishes for 24-hours. Following 24 hour treatment with FTY720, the media were replaced with fresh complete media. After 10 days of incubation, the cells were fixed and stained with crystal violet. Colonies with above 50 cells were counted for each dish.

### Apoptotic assays

To measure apoptosis in FTY720-treated cells, Annexin V staining, DNA laddering and Caspase activity assays were performed. For Annexin V staining, cells were harvested, stained with FITC-conjugated Annexin V and propidium iodide (PI; FITC Annexin V Apoptosis Detection kit I, BD Pharmigen^TM^, USA), and analysed by a BD FACS Canto II flow cytometer (BD Biosciences, CA, US). The percentage of early apoptotic (Annexin V-positive and PI-negative) and late apoptotic (Annexin V-positive and PI-positive) cells were determined using BD FACSDiva^TM^ (BD Biosciences, CA, US). For DNA laddering, DNAs from FTY720-treated cells were extracted using an apoptotic DNA ladder extraction kit (Biovision, California, USA) and subjected to electrophoresis. Caspase-3/7, 8 and 9 activities of treated cells were measured using a Caspase-Glo® 3/7, 8 and 9 assay kit (Promega, USA), according to the manufacturer’s instructions.

### Western Blotting analysis

Following treatment with FTY720 in complete media, cells in 100-mm dishes were lysed in ice-cold RIPA lysis buffer containing protease inhibitors (cocktail set III; Calbiochem, Merck Millipore) and phosphatase inhibitors (Halt phosphatase inhibitor cocktail; Thermo Fisher Scientific, Waltham, USA). The protein lysates were collected and subjected to western blot analysis. The primary antibodies used were anti-Akt and anti-phospho-Akt (Ser473) (1:1000 dilution; Cell Signalling, USA), anti-ERK and anti-phospho-p44/42 MAPK (Erk1/2) (Thr202/Tyr204) (1:1000 dilution; Cell Signalling, USA), and secondary antibodies were HRP-conjugated goat anti-rabbit IgG or goat anti-mouse IgG (Sigma-Aldrich). The protein bands were detected with chemiluminescent reagent, Western Bright TM Sirius (Advansta, CA, USA) and viewed in an Oddyssey FC Imaging system (Licor Biosciences, Cambridge, UK).

### Statistical Analysis

Mann-Whitney’s U test was performed to evaluate the statistical difference in the mRNA levels between the normal oral and OSCC tissues. The association of the gene expression with cancer stage was determined using Pearson chi-square test. Kaplan Meier survival analysis was used to estimate the survival function for gene expression. For *in vitro* experiments, unpaired t-test (to compare two means) or one-way ANOVA with post-hoc Dunnett’s test (to compare means against a single mean) were performed.

## Additional Information

**How to cite this article**: Patmanathan, S. N. *et al.* Aberrant expression of the S1P regulating enzymes, SPHK1 and SGPL1, contributes to a migratory phenotype in OSCC mediated through S1PR2. *Sci. Rep.* **6**, 25650; doi: 10.1038/srep25650 (2016).

## Supplementary Material

Supplementary Information

## Figures and Tables

**Table 1:** Clinicopathological characteristics of OSCC samples.

Characteristics	Tumour (n = 52)(%)	Normal (n = 5)(%)
Gender		
Male	18 (34.6)	3 (60.0)
Female	34 (65.4)	2 (40.0)
Age (years)		
≤50	17 (32.7)	5 (100.0)
>50	35 (67.3)	0 (0.0)
Stage		
Early (I and II)	19 (36.5)	N/A
Advanced (III and IV)	33 (63.5)	N/A
Status		
Alive	26 (50)	N/A
Deceased	26 (50)	N/A
