# Supplementary material for: Aberrant expression of the S1P regulating enzymes, SPHK1 and SGPL1, contributes to a migratory phenotype in OSCC mediated through S1PR2
Source: Sci Rep. 2016 May 10;6:25650. doi: 10.1038/srep25650 (PMC4861980; doi:10.1038/srep25650)
Supplement: Supplementary Information [file srep25650-s1.pdf]

Aberrant expression of the S1P regulating enzymes, SPHK1 and SGPL1, contributes to a migratory phenotype in OSCC mediated through S1PR2

Sathya Narayanan Patmanathan, Steven P. Johnson, Sook Ling Lai, Suthashini Panja Bernam, Victor Lopes, Wenbin Wei, Maha Hafez Ibrahim, Federico Torta, Pradeep Narayanaswamy, Markus R. Wenk, Deron R. Herr, Paul G. Murray, Lee Fah Yap and Ian C. Paterson

Supplementary Figures:

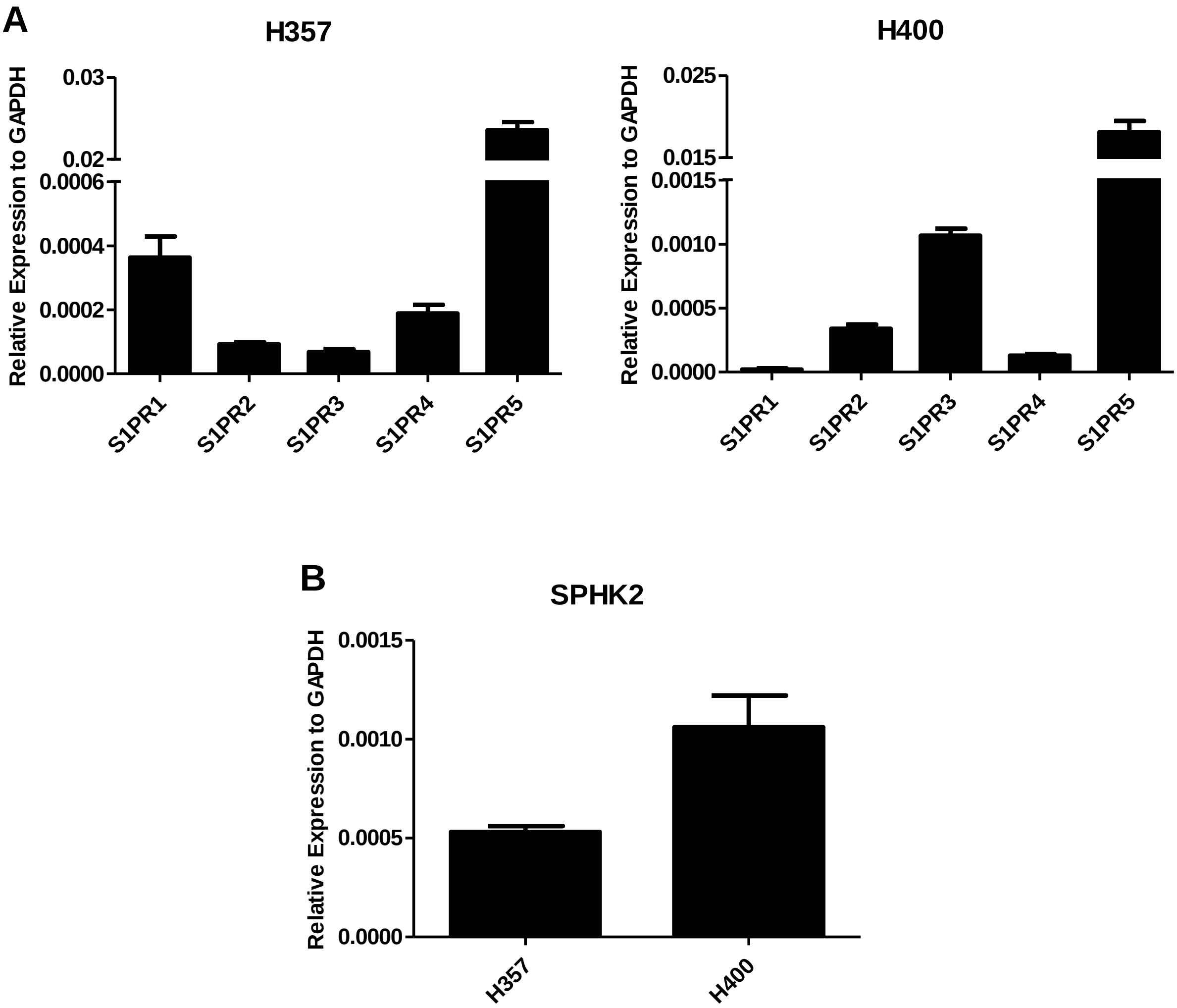

Figure S1. The mRNA expression of S1PR1-5 (A) and SPHK2 (B) (normalised to GAPDH expression) in H357 and H400 as determined by QPCR. Results are shown as mean ± SD values of triplicates.

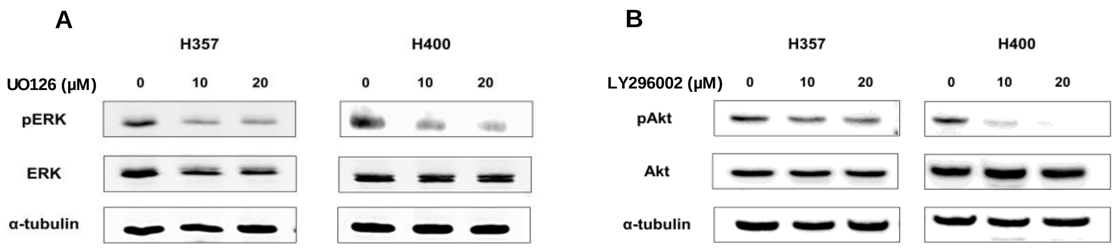

Figure S2. Western blotting was performed following treatment of H357 and H400 with UO126 (A) and LY296002 (B) for 24 hours.

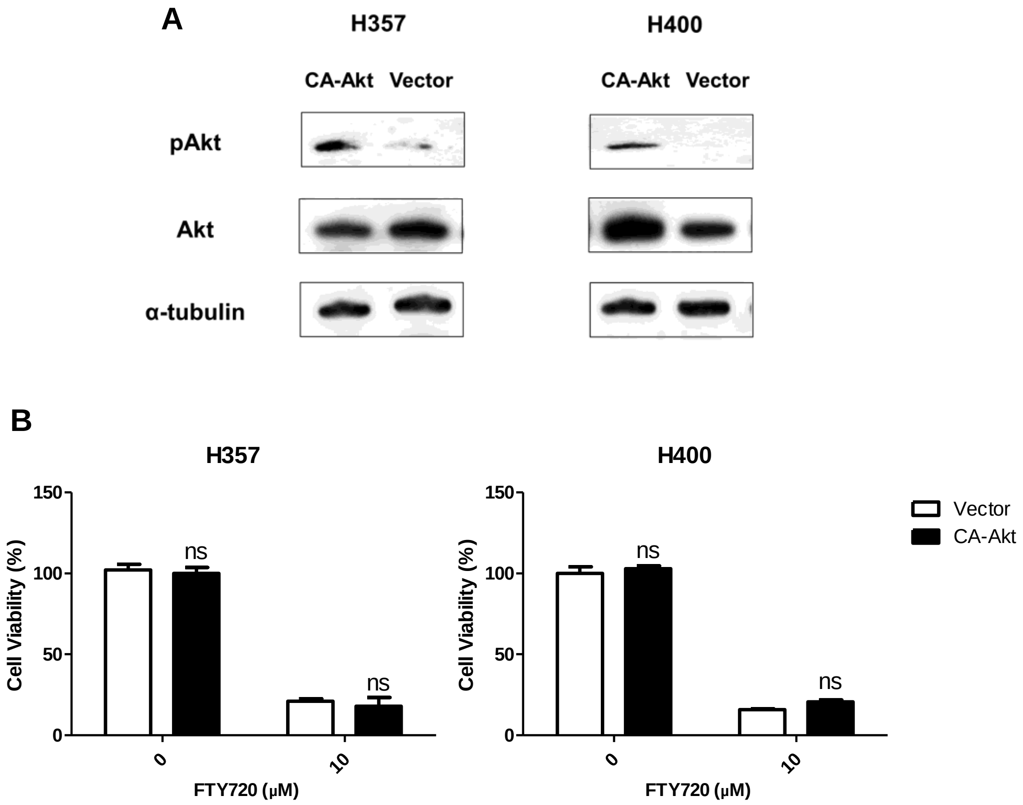

Figure S3. H357 and H400 cells were transfected with pcDNA 3.1 (vector) or constitutively active Akt (CA-Akt) plasmids for 48 hours. The cells were then treated with vehicle control (DMSO) or FTY720 (10μM) for 24 hours. Western blot was carried out to confirm expression of CA-Akt. (B) Graphs show the results expressed as a percentage of cell viability in vehicle control (=100%) as determined by MTT assay. Bars, mean; error bars, SD of triplicates. ns = not-significant (unpaired t-test).

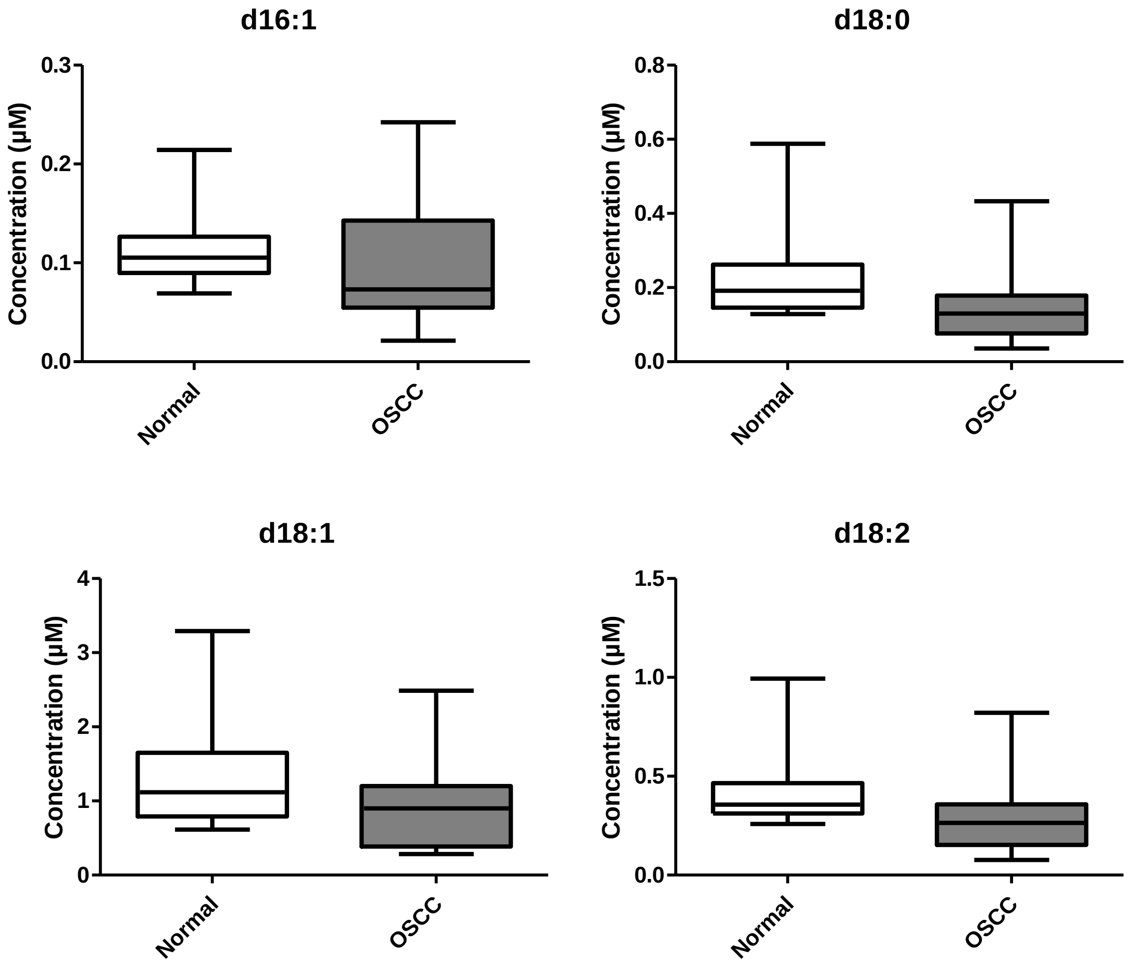

Figure S4. Box plots show concentration of different S1P species in the plasma of normal (N=12) or OSCC patients (N=12) as determined by the LC-MSMS analysis.

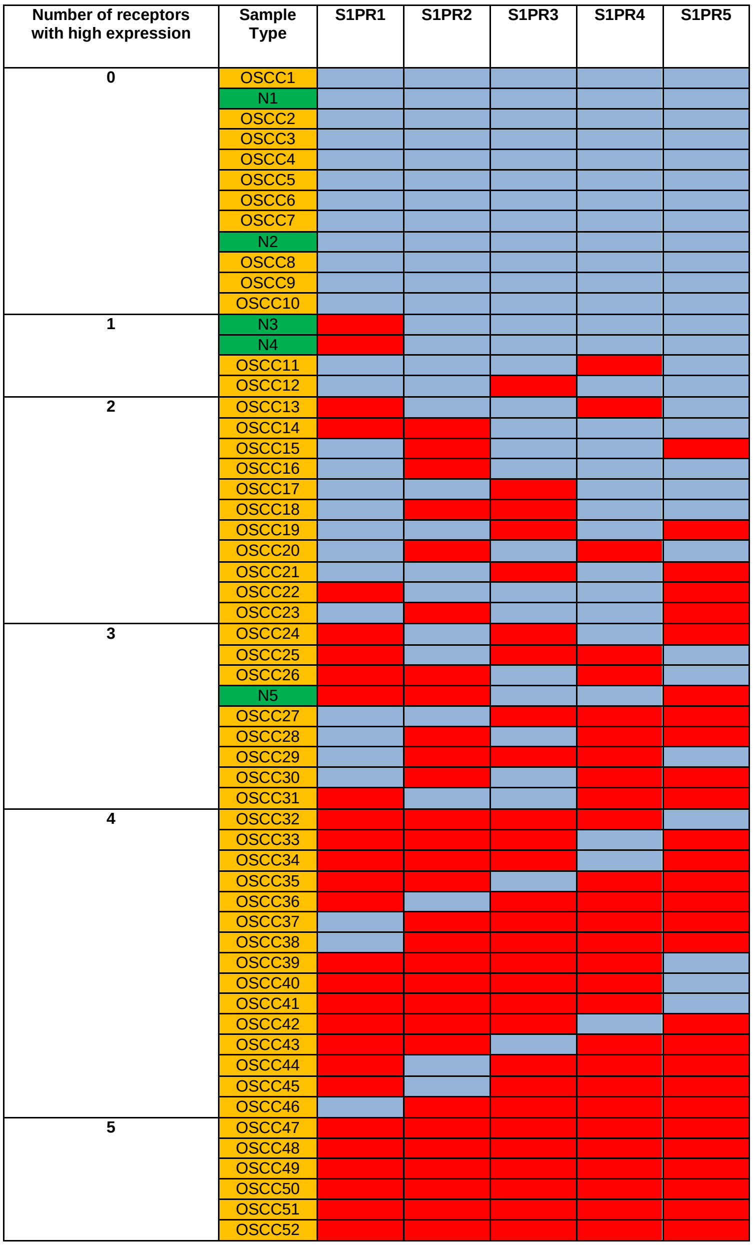

Figure S5. All the tissues samples were classified into two groups based on expression levels of each S1PR mRNA. Samples with normalised expression (NE) values (NE= $2^{-\Delta\Delta CT}$; $\Delta\Delta CT$ obtained from qPCR analysis) lower than the median NE of the samples were assumed to have low expression (indicated as blue box), whereas those with higher than the median NE were grouped into high expression (indicated as red box). Table shows samples arranged in order based on the number of S1PR with high expression. Normal and OSCC samples are indicated in green and orange respectively.

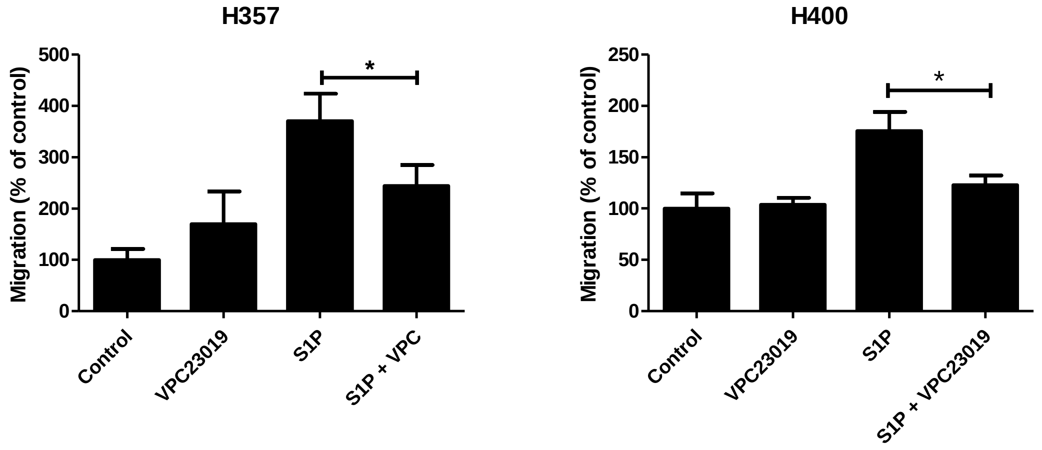

Figure S6. Transwell assays were performed in the presence or absence of 5µM S1P and/or 5µM VPC23019 in the lower chamber. Results are expressed as percentage of migrated cells in the untreated control (=100%) ± SD. * = $\rho<0.05$ (unpaired T-test).

Table S1. List of genes related to S1P metabolism and signaling used in HG-Focus array.

ABCA1	DEGS1	MAPK1	S1PR4
ABCB1 /// ABCB4	DGKA	NEU3	S1PR5
ABCC1	DGKD	PLD1	SGPL1
ABCC9	DGKE	PLD2	SGPP1
AGPAT1	DGKG	PPAP2B	SLC35A2
AGPAT2	DGKI	PPAP2C	SMPD1
AGPAT3	DGKQ	PRKCA	SMPD2
AGPAT4	EGFR	PRKCB	SPHK1
ARSA	ENPP2	PRKCD	SPTLC2
ARSB	GAL3ST1	PRKCE	TNF
ARSD	GALC	PRKCG	TNFRSF10B
ARSE	GBA /// GBAP1	PRKCH	TNFRSF10C
ASAH1	GLA	PRKCI	UGCG
B4GALT6	GLB1 /// TMPPE	PRKCQ	UGT8
CDS1	IGFBP3	S1PR1	KDSR
CERS1 /// GDF1	IL1B	S1PR2	
